# Blood Coagulation Activities of Cotton–Alginate–Copper Composites

**DOI:** 10.3390/md21120625

**Published:** 2023-11-30

**Authors:** Zdzisława Mrozińska, Michał Ponczek, Anna Kaczmarek, Maciej Boguń, Edyta Sulak, Marcin H. Kudzin

**Affiliations:** 1 Łukasiewicz Research Network—Lodz Institute of Technology, 19/27 Marii Sklodowskiej-Curie Str., 90-570 Lodz, Poland; 2 Department of General Biochemistry, Faculty of Biology and Environmental Protection, University of Lodz, 90-236 Lodz, Poland

**Keywords:** alginic acid, antifungal activity, antimicrobial activity, blood coagulation, composite, copper, cotton-based materials, composite, activated partial thromboplastin time, polymer functionalization, prothrombin time, thrombin time

## Abstract

Alginate-based materials have gained significant attention in the medical industry due to their biochemical properties. In this article, we aimed to synthesize Cotton–Alginate–Copper Composite Materials (COT-Alg^(−)^Cu^(2+)^). The main purpose of this study was to assess the biochemical properties of new composites in the area of blood plasma coagulation processes, including activated partial thromboplastin time (aPTT), prothrombin time (PT), and thrombin time (TT). This study also involved in vitro antimicrobial activity evaluation of materials against representative colonies of Gram-positive and Gram-negative bacteria and antifungal susceptibility tests. The materials were prepared by immersing cotton fibers in an aqueous solution of sodium alginate, followed by ionic cross-linking of alginate chains within the fibers with Cu(II) ions to yield antimicrobial activity. The results showed that the obtained cotton–alginate–copper composites were promising materials to be used in biomedical applications, e.g., wound dressing.

## 1. Introduction

Cotton is a natural fiber composed mainly of pure cellulose, and it is commonly applied in the apparel and textile industry [1,2]. This is due to its favorable properties, including softness, mechanical strength, elasticity, and water affinity [1]. Moreover, it is a low-cost and biodegradable material [1]. Apart from that, cotton exhibits a set of properties that are very desirable for medical and biomedical applications, including a porous structure, a large surface area, and gas permeability [2]. Thus, it is widely employed for the production of health care textiles [2]. The use of cotton for medical purposes may be divided into two categories: external and internal biomaterials [2]. The first group includes medical clothing (such as gowns, masks, uniforms, and caps), surgical covers and drapes, as well as beddings [2]. The second one constitutes products used for tissue engineering, dental and surgical applications, drug delivery systems, as well as wound dressings [2].

As far as wound dressings are concerned, cotton gauze is one of the most popular and widely used wound dressings [2]. It is a dry wound dressing with a fibrous structure, and it has the ability to absorb the wound exudate [2]. However, it saturates very quickly and requires frequent changing, which is not cost effective [2]. Another drawback of a cotton gauze is its adherence to the wound, which causes damage to the tissue during its removal [2]. Moreover, cotton does not exhibit antimicrobial activity; thus, it is prone to microbial contamination, leading to the development of infection [1,2]. Therefore, cotton gauze is mainly applied as a secondary dressing or for wounds with low exudate, while its use for chronic, highly exuding wounds, such as burns or ulcers, is highly limited [2].

Therefore, researchers are currently focused on the development of more advanced wound dressings based on multifunctional textiles [1,2]. These attempts involve the surface modification of cotton (for example, using different biopolymers, such as chitosan or alginate), in order to overcome the abovementioned disadvantages of traditional cotton gauze wound dressings [1,2,3,4].

**Figure 1:**
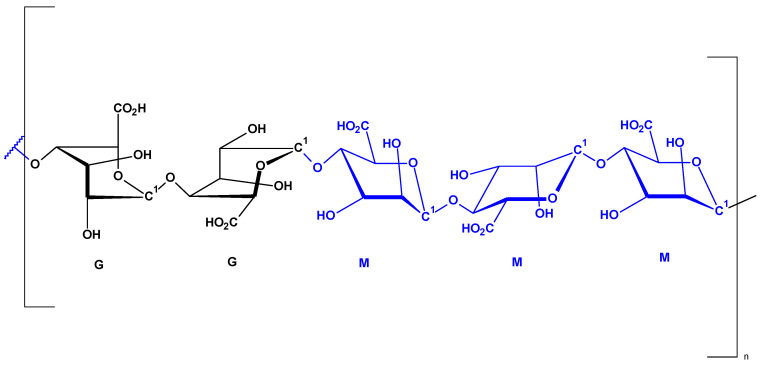
Structure of GGMMM fragment of alginic acid (M—β-D-Mannuronate and G—α-L-guluronate).

Alginate is a polysaccharide of natural origin usually derived from brown seaweed but also from bacteria [5,6,7,8,9,10,11,12,13,14,15,16,17,18]. It is a hydrophilic, water-soluble anionic biopolymer containing blocks of (1,4)-linked-β-D-mannuronate (M) and α-L-guluronate (G) residues [5,7,8,9,10,11,12,13,14,15,16,17,18,19] (Figure 1).

Alginate is well known from its remarkable properties, such as chemical versatility, biocompatibility, biodegradability, non-immunogenicity, non-toxicity, and biological activity [5,7,8,9,10,11,12,13,14,15,16,17,18,20,21]. Moreover, it is the second most abundant biopolymer available (after cellulose), and its extraction and processing costs are relatively low [7]. Finally, alginate has a very good cross-linking capability, and it is easy to process and modify [8,14,17,21]. This is due to its distinct physical and chemical structure, including, mainly, the presence of free hydroxyl and carboxyl groups, as well as its sol-to-gel transformation ability [5,8]. This not only allows it to develop alginate-based composites with specific chemical and physical properties but also makes alginate a perfect material used to finish textiles (for instance, cotton) [8]. Currently, there are multiple different techniques used for textile functionalization using alginate, such as nanocomposite coating, ionic cross-linking coating, and layer-by-layer coating [8].

Due to the abovementioned outstanding properties, alginate is gaining a lot of interest in terms of its biological and medical applications, mainly for tissue engineering and wound healing [5,6,7,8,9,10,11,12,13,14,15,16,17,18,19,20,21,22,23]. It is believed that alginate-based wound dressings provide an appropriate drainage of wound exudates and prevent the entry of pathogenic bacteria [9,10,11,12,13]. Alginate’s high content of water allows it to ensure the proper moist environment [5], which in turn helps to keep the optimal pH [18,24]. This is of the utmost importance because it was proven that a wet environment is considerably more effective in terms of wound healing [2]. Furthermore, alginate exhibits a unique hemostatic effect owing to its gel-forming ability and high adsorption capacity [19]. According to the literature, the water absorption rate of an alginate fiber is equal to 2.2 times its mass [15]. Alginate-based dressings absorb blood and body fluids and form hydrogels, which adhere to the surface of the wound, leading to the compression of the bleeding wound [15,25]. Additionally, due to the presence of mannuronic (M) or guluronic (G) groups with high calcium (Ca) content, alginates serve as donors of calcium ions [26], which enter the wound as a result of the ion exchange reaction with sodium ions present in blood [15,20]. Calcium ions are responsible for the stimulation of clotting factors (VII, IX, X), activation of the coagulation cascade reaction, and platelet aggregation [15,22]. It was also reported, that depending on its structure and M or G group composition, alginate may influence blood coagulation to different extents [22]. Moreover, some studies suggest that alginate promotes the growth of the new epidermis [5]. Thus, alginate-based materials are considered to accelerate the process of hemostasis and wound healing [15,18,21,23,24].

Another additional factor that has to be taken into account when it comes to effective wound dressing materials is the inhibition of bacterial growth. According to some studies, the alginate-based materials exhibit antimicrobial activity [3,18,27]. For instance, Rafiq et al. pointed out that alginate interacts with a bacterial outer surface through negative charges and, as a consequence, causes the disruption of bacteria [5,28]. What is more, alginates were demonstrated to kill microorganisms as they block the nutrient transportation by forming a viscous layer around them [5]. Nevertheless, the observed antimicrobial effect is insufficient in terms of wound healing. Thus, alginate composite materials composed of various additives are investigated with the aim of enhancing different properties, including not only antibacterial activity, but also cell viability and adhesion, as well as blood clotting within hemostasis. Among the different antimicrobial agents, copper is attracting growing attention.

On the one hand, copper in its ionized form as a trace element is a transition metal necessary for the proper functioning of the human organism, being a cofactor of many copper enzymes participating in redox reactions, and, on the other hand, the biocidal properties of copper and its complexes have been well documented in the literature [29,30,31,32,33,34,35,36,37]. Furthermore, despite the high susceptibility of microorganisms to copper, human tissues do not exhibit sensitivity towards it, as it is needed for their proper functioning [38,39,40]. Additionally, copper is also significantly cheaper than silver, which is a very popular antimicrobial agent [32,41]. Therefore, copper is considered to be a very effective additive used for the antimicrobial functionalization of alginate-based materials as well as textiles [42,43,44,45,46,47,48]. Moreover, copper plays an important role in the wound-healing process [47,48,49,50,51]. This is due to the stimulation of the activity of copper-dependent enzymes, proteins, and polysaccharides, which results in the positive influence on the matrix remodelling, cell proliferation, and reepithelization [49,50,51]. Furthermore, copper takes part in the generation of the vascular endothelial growth factor and thus affects the angiogenesis [49,50,52,53]. In addition, copper is responsible for the stabilization of fibrinogen and collagen, and it increases the expression of integrin [40,50,54].

The main biological aim of the current study is to test if the new cotton–alginate–copper-based materials with optimally selected metallic component concentration have any detrimental effect on blood plasma coagulation as the first step during wound care, while maintaining antimicrobial effects. Therefore, aPTT and PT measurements have been carried out. aPTT enables the study of the intrinsic (contact) blood plasma coagulation pathway and reveals the time of fibrin clot formation after activation on a negatively charged surface (in the presence of kaolin) where contact factors (XII, XI), plasma prekallikrein (PK), and high molecular kininogen (HK) initiate the blood coagulation cascade. PT is used to study blood clotting triggered by extrinsic pathway (tissue factor—thromboplastin, calcium ions), where tissue factor (TF) activates factor VII, thus leading to fibrin formation, when a prothrombinase complex is formed [55]. In this way, both pathways of the plasma component of blood coagulation can be tested using aPTT and PT.

## 2. Results and Discussion

### 2.1. Preparation of Composite Material

**Figure 2:**
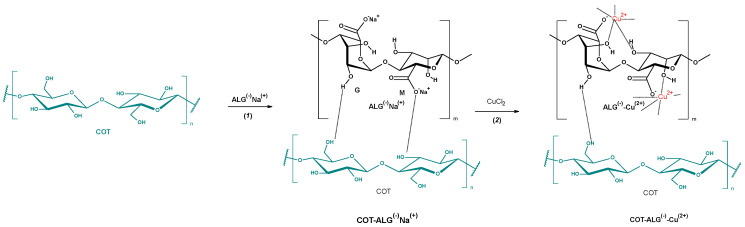
The schematic diagram of the procedures for surface modification of cotton material using alginate.

The cotton fabric samples were modified through a dip-coating, two-step method, based on our previous work related to the modification of poly(lactic acid) fiber materials [40,56]. Samples of cotton fabric (COT) were impregnated in a homogeneously dispersed polysaccharide solution for 1 min (0.5%—COT-ALG^(−)^Na^(+)^-1 and 5%—COT-ALG^(−)^Na^(+)^-2) and then immediately transferred into two different aqueous solutions of copper (II) chloride and re-immersed for 1 min (COT-ALG^(−)^Cu^(2+)^-1—5% CuCl_2_ solution, COT-ALG^(−)^Cu^(2+)^-2 in 10% CuCl_2_) (Figure 2). Then, the alginate composites were squeezed and dried for 6 h at 40 °C to constant weight. For the biological and biochemical comparative research, we modified cotton fabric samples using analogous concentrations of copper solution without alginate modification.

Table 1 presents abbreviations of the cotton samples used for the investigation. Because the parts of these abbreviations contain chemical formulas, we used COT for cotton instead of CO/Co (CO and/or Co can be confused with carbon oxide or cobalt).

### 2.2. Chemical and Structural Characterization

#### 2.2.1. Fourier Transform Infrared Spectroscopy

**Figure 3:**
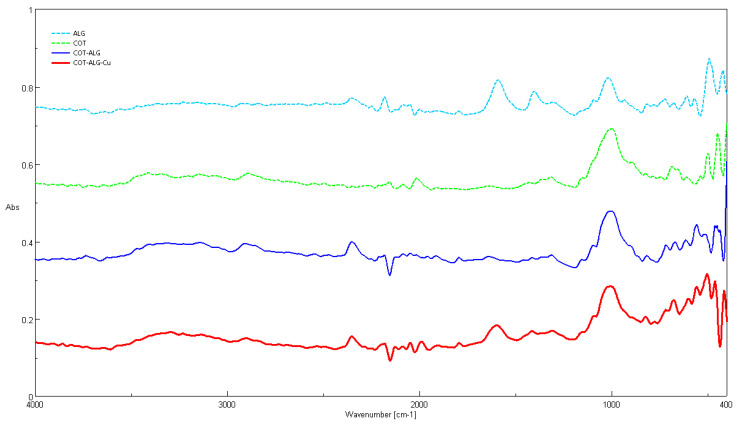
Fourier transform infrared spectroscopy (FTIR) spectra of: alginic acid sodium salt (ALG); cotton fabric (COT); cotton–alginate composite (COT-ALG^(−)^Na^(+)^(1%)); cotton–alginate–copper composite (COT-ALG^(−)^Cu^(2+)^(0.15)).

Characteristic FTIR bands ν (cm^−1^) determined for COT fabrics, alginate salts, and copper composites (ALG^(−)^Na^(+)^, ALG^(−)^Cu^(2+)^ and COT-ALG^(−)^Cu^(2+)^) are presented in Figure 3. Table 2 lists infrared spectroscopy absorptions by frequency regions in the spectral range of 4000–400 cm^−1^ of the samples, with the designation of chemical bonds. The comparison between the FTIR spectra of the alginic acid sodium salt and cotton fabric indicates the presence of main absorption bands in the cotton–alginate–copper composite (COT-ALG^(−)^Cu^(2+)^), which is confirmed by the data from the literature [57,58,59,60,61,62,63,64,65].

#### 2.2.2. Flame Atomic Absorption Spectrometry

Determination of the Cu concentration in the cotton–alginate–copper composite samples was performed by means of FAAS spectrometry, and the results are shown in Table 3.

The results of the determination of the copper concentration in the composites show that the metal content in complex materials depends on the variant of the applied modifier dip-coating solution and the type of solution of CuCl_2_ (Table 1). The higher concentration of the used copper (II) chloride solutions (10%) resulted in the higher content of the metal in the cotton–alginate composite (17.51 g/kg) and cotton sample (COT-Cu^(2+)^(0.30)—19.19 g/kg), and the lower concentration of the Cu solution (5%) gave a relatively lower content of Cu in the sample (COT-Alg^(−)^Cu^(2+)^(0.15)—9.35 g/kg/COT-Cu^(2+)^(0.16)—10.06 g/kg). Additionally, the FAAS spectrometry measurements also indicated that the distribution of copper in the material is quite uniform (approximately 4.5%).

#### 2.2.3. Morphology and Elemental Analysis

##### Microscopy Analysis

**Figure 4:**
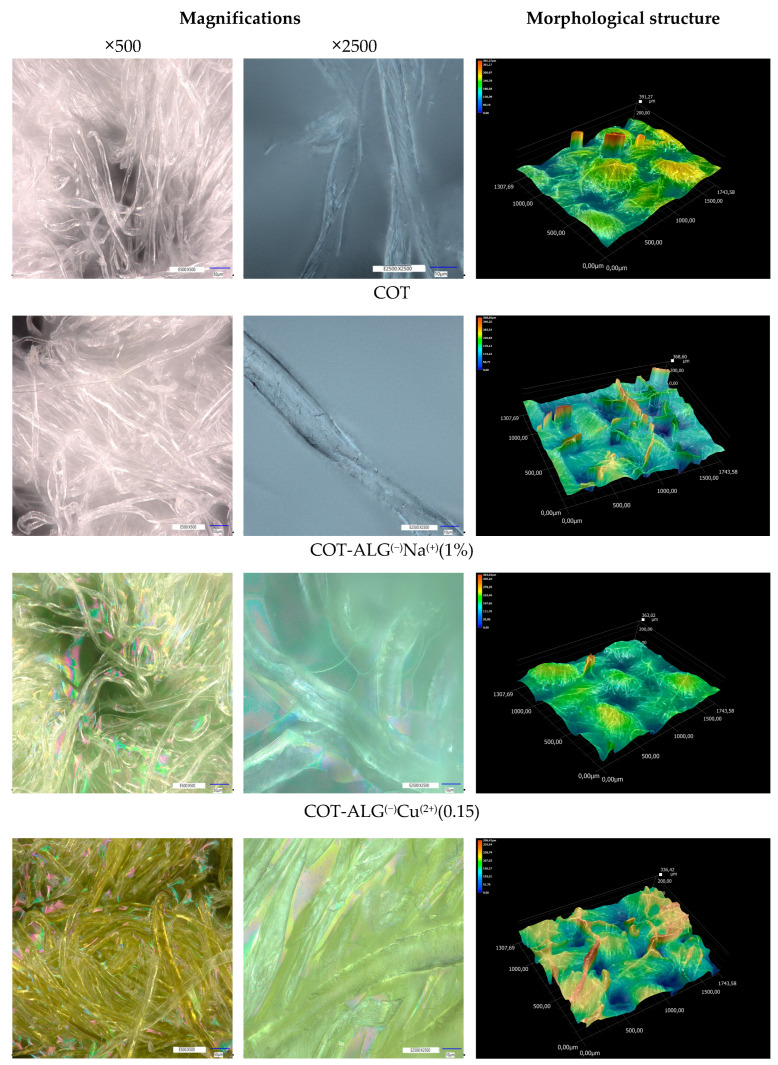
Optical microscopy images (magnifications: ×500, ×2500) and morphological visualization of surface structure of samples before and after modification processes.

The analysis of changes in the morphological and elemental structure of the surface and fibers of cotton fabric after modification was carried out using a digital microscope with the laser-based elemental analyzer. Figure 4 shows microscope images of the samples before and after modification processes under the magnifications equal to ×500 and ×2500. Table 4 shows the quantitative analysis of the content of elements on the fiber surface based on the laser-based elemental analyzer.

As far as the morphology of the investigated samples is concerned, it may be ob-served that the modification of the samples with alginate (COT-Alg^(−)^Na^(+)^) and copper (II) chloride (COT-Alg^(−)^Cu^(2+)^) caused some noticeable changes. The two-step dip-coating modification led to the formation of the film interconnecting the cotton fibers, which is especially apparent for the COT-Alg^(−)^Cu^(2+)^(0.28) sample. At the same time, the pores become less visible, which may be due to the fact that the void spaces were filled with the alginate and copper. This was also confirmed by the 3D microscopic images showing the morphological structure of the samples, which revealed that the modification of the cotton resulted in the smoother surfaces, i.e., the height difference between the lowest and the highest points on the surface decreased. Similar results were observed in our previous study for the PLA nonwovens modified with sodium alginate and zinc (II) chloride [56].

The observations under the higher magnification (×2500) revealed that the modification of the cotton samples with alginate and CuCl_2_ did not cause any significant damage to the fibers.

The laser-based elemental analysis (Table 4) of the surface of the investigated samples confirmed the presence of the elements, which were the structural components of both cellulose and alginate, i.e., oxygen, carbon, and hydrogen. In the case of the samples modified with copper (II) chloride, the copper was also present on the surface, and its concentration increased with the increasing concentration of the CuCl_2_ in the solution used for the modification.

As far as the quantitative analysis is concerned, it was observed that in the case of the sample modified with sodium alginate only, the concentration of hydrogen increased, while the concentration of carbon decreased. In the case of samples modified with copper (II) chloride, the relative content of carbon and oxygen slightly decreased due to the appearance of copper on the surface. The concentration of coper increased from 4 to 13 wt. %. with the increase in the CuCl_2_ concentration in the solution from 5 to 10%. The increase in the copper concentration is in agreement with the results obtained through FAAS, which also revealed the increase in the copper content for the sample COT-ALG^(−)^Cu^(2+)^(0.28). The analysis of the data also revealed substantial differences in the bulk and surface elemental content. Thus, for cotton, the carbon concentration was over 20% lower for the surface than in bulk, whereas hydrogen is over 30% higher. These data suggest that surface carbon atoms were covered by hydrogens. Surface carbon contents in COT-ALG^(−)^Cu^(2+)^ composites were lower than for unmodified cotton due to lower carbon contents in ALG^(−)^Na^(+)^ in comparison with COT. Lowering of the surface contents of carbon and oxygen during the increase in the copper charge in COT-ALG^(−)^Cu^(2+)^ resulted from the increase in the copper content as well as the masking effect of the smaller atoms, i.e., C and H, by large copper atoms. It is worth noting that the surface content of copper atoms is 4–7.5 times higher than in bulk.

##### Specific Surface Area

The total pore volume and specific surface area of the cotton sample (COT) and copper–alginate composites (COT-ALG^(−)^Na^(+)^, COT-ALG^(−)^Cu^(2+)^) are presented in Table 5.

The modification of cotton material with alginate (COT-ALG^(−)^Na^(+)^) and copper (II) chloride (COT-ALG^(−)^Cu^(2+)^) leads to the lowering of the specific surface area and total pore volume (BET). The specific surface area of the cotton material (COT) sample was equal to 0.7359 m^2^/g. The modification of cotton fabric in the solution of alginic acid sodium salt resulted in the decrease of the specific surface area to 0.6191 m^2^/g (COT-ALG^(−)^Na^(+)^(1%)). With the increase in the concentration of the sodium alginate, the specific surface area decreased even further to 0.5577 m^2^/g. Similarly, the subsequent immersion in a copper(II) chloride solution (COT-ALG^(−)^Cu^(2+)^(0.15)/(0.28)) also resulted in the lowering of the specific surface area; however, the observed changes were not significant. The higher the concentration of Cu, the lower the specific surface area (0.6042 and 0.5931 m^2^/g, respectively).

At the same time, a significant decrease in the total pore volume for the modified samples may be observed from 7.408 × 10^−3^ cm^3^/g to 3.095 × 10^−3^–2.840 × 10^−3^ cm^3^/g. In the case of the cotton samples modified with the solution of alginic acid sodium salt, the higher the concentration of sodium alginate, the lower the total pore volume observed. Further modification with copper (II) chloride also caused a decrease in the total pore volume; however, the change was not as significant. A similar trend may be observed as was noted earlier, i.e., a higher concentration of Cu leads to a slightly lower total pore volume.

The observed decrease in the specific surface area and total pore volume is probably due to the fact that the pores present in the samples were filled with the sodium alginate and copper used for the modification. This is in agreement with the fact that increasing sodium alginate and copper concentration resulted in the lowering of the total pore volume and specific surface area. The decrease in the porosity caused the decrease of the specific surface area as a consequence. The obtained results are in agreement with the morphological analysis of the surface of the investigated samples, which revealed that the pores became less visible due to the performed modifications, and the observed surfaces became smoother as a result of the formation of the film interconnecting the cotton fibers.

In conclusion, the most significant change in the specific surface area and total pore volume was observed for the modification with a 0.5% solution of sodium alginate. A further increase in the sodium alginate concentration and the subsequent immersion in CuCl_2_ solution, as well as an increase in the concentration of Cu, resulted only in a slight change in the observed parameters.

**Figure 5:**
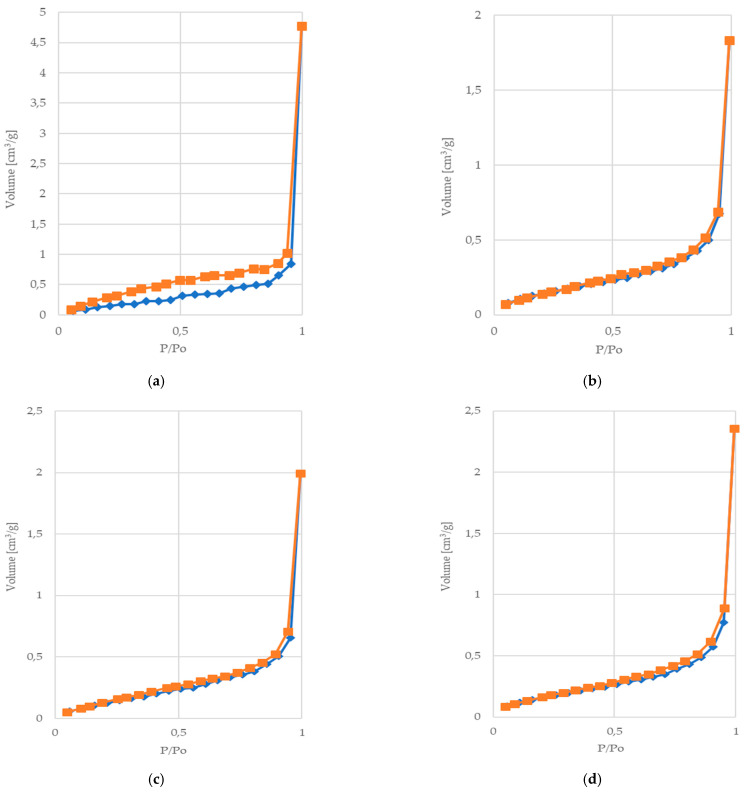
Isotherms of nitrogen adsorption/desorption at 77 K in the various types of materials studied in this work: (**a**) COT; (**b**) COT-ALG^(−)^Na^(+)^(1%); (**c**) COT-ALG^(−)^Cu^(2+)^(0.16); (**d**) COT-ALG^(−)^Cu^(2+)^(0.28). Lines are results of approximation.

Figure 5 presents the N_2_ adsorption–desorption isotherms obtained for the investigated samples. For all of the samples, the amount of adsorbate increases exponentially with the growing pressure. Because the obtained absorption isotherms resemble the hyperbolic graph with no distinct “knee” formed, they can be classified as type III according to the IUPAC classification [65,67]. The type III isotherm is characterized by its non-porous and/or macroporous materials, and it is typically observed for relatively weak interactions between the adsorbent and adsorbate [65,67]. This type of isotherm is associated with low uptake at a low concentration followed by the significant increase in sorption at higher vapor concentration [65,67]. The lack of a distinctive “knee” associated with the formation of a well-defined monolayer indicates that the sorption occurs fully according to the multilayer mechanism [65,67].

The presence of the hysteresis loop, i.e., the difference between the adsorption and desorption isotherms, is usually related to the capillary condensation in mesopore structures [65,67,68]. It occurs due to the fact that capillary condensation takes place at a higher relative pressure than capillary evaporation [65]. In the case of the obtained isotherms, the type H3 hysteresis loop may be observed for all of the samples. This type of hysteresis loop occurs when the macrospores are not fully filled with pore condensate, and it is typical of slit-shaped pores [65,67,68]. The size of the observed loops decreases significantly for the modified samples, which may be associated with the observed lower total pore volume.

#### 2.2.4. Air Permeability

Determination of the air permeability of cotton–alginate–copper composites was carried out according to the EN ISO 9237:1998 standard [69], and all results are shown in Table 6.

The results indicate that alginate modification decreased the air permeability of all coated samples. The higher concentration of alginate used during the modification (COT-ALG^(−)^Na^(+)^(9.7%)) reduced the permeability of the composite (from 278 to 39.3 mm/s). At the same time, the permeability was not significantly affected by the modification with copper chloride (COT-ALG^(−)^Cu^(2+)^: 283/286 mm/s). The samples modified with different concentration of CuCl_2_ had approximately similar result of air permeability (COT-ALG^(−)^Cu^(2+)^(0.15)/(0.28): 283/286 mm/s). The decrease in air permeability for the modified samples may be associated with the lowering of the total pore volume due to the filling of pores with the sodium alginate and copper. The most significant change in the air permeability corresponds to the lowest value of the total pore volume.

#### 2.2.5. Tensile Testing

The results of the tensile strength measurements (durability for stretching and relative elongation at maximum load) of the cotton–alginate–copper composites are listed in Table 7.

The results show the increase in tensile strength after the alginate modification of the cotton material (COT-ALG^(−)^Na^(+)^(1%): 510/370 and COT-ALG^(−)^Na^(+)^(9.7%): 580/410 kN/m) in comparison with the unmodified sample (COT: 490/380 kN/m). The higher the concentration of sodium alginate, the higher the tensile strength. The subsequent immersion in CuCl_2_ did not influence the tensile strength. Similarly, there was a slight increase in the relative elongation for the sample modified with 0.5% solution of sodium alginate; however, it decreased with the increase in the sodium alginate concentration (Table 6). Further modification with CuCl_2_ did not affect the relative elongation.

### 2.3. Biological and Biochemical Properties

#### 2.3.1. Antimicrobial Activity

**Figure 6:**
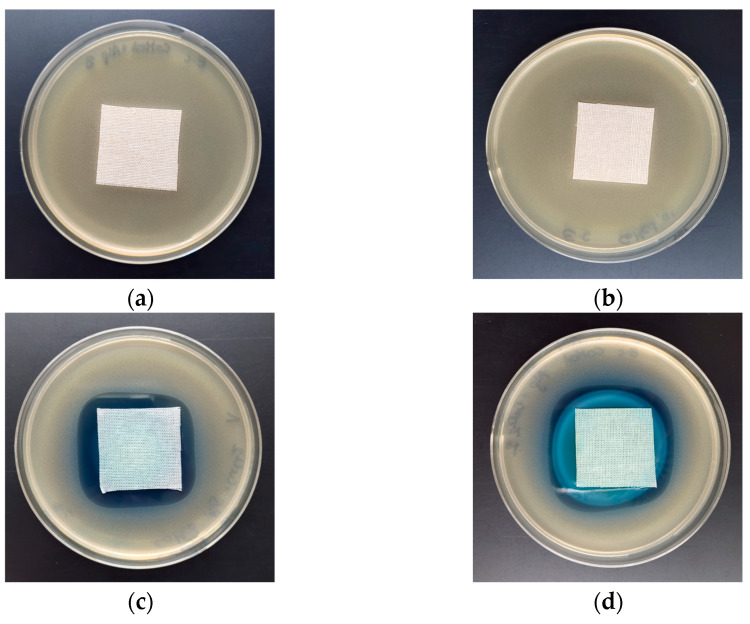
Tests of antimicrobial activity of alginate composites against *E. coli;* inhibition zones of bacterial growth in Petri dishes: (**a**) COT; (**b**) COT-ALG^(−)^Na^(+)^(1%); (**c**) COT-ALG^(−)^Cu^(2+)^(0.15); (**d**) COT-ALG^(−)^Cu^(2+)^(0.28).

**Figure 7:**
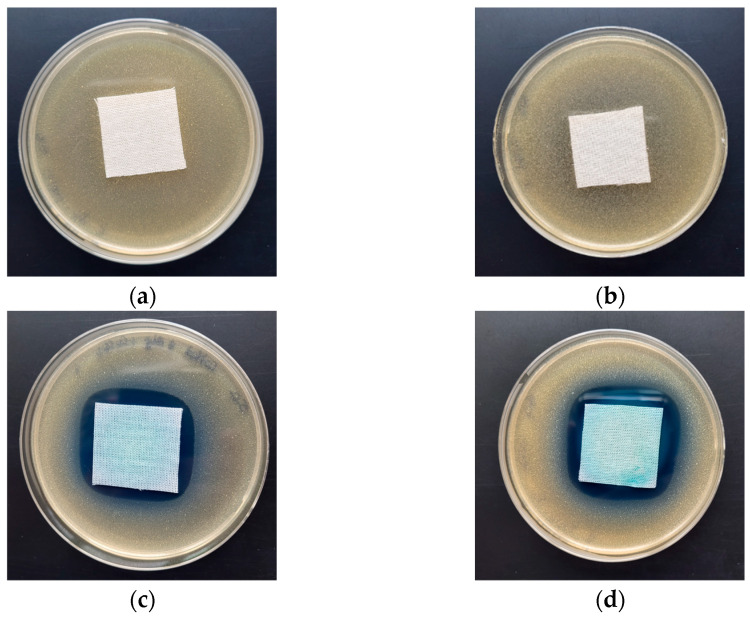
Tests of antimicrobial activity of alginate composites against *S. aureus;* inhibition zones of bacterial growth in Petri dishes: (**a**) COT; (**b**) COT-ALG^(−)^Na^(+)^(1%); (**c**) COT-ALG^(−)^Cu^(2+)^(0.15); (**d**) COT-ALG^(−)^Cu^(2+)^(0.28).

**Figure 8:**
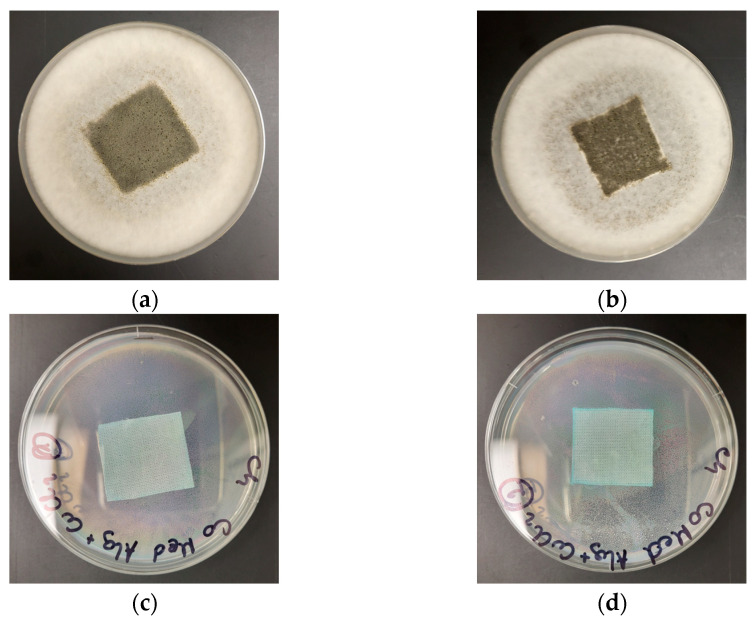
Tests of antimicrobial activity of alginate composites against *Ch. globosum;* inhibition zones of bacterial growth in Petri dishes: (**a**) COT; (**b**) COT-ALG^(−)^Na^(+)^(1%); (**c**) COT-ALG^(−)^Cu^(2+)^(0.15); (**d**) COT-ALG^(−)^Cu^(2+)^(0.28).

The antimicrobial activity of COT-ALG^(−)^Cu^(2+)^ composites and COT-Cu^(2+)^ samples was investigated through the disk diffusion method using Gram-negative (*Escherichia coli*, *Pseudomonas aeruginosa*) and Gram-positive (*Staphylococcus aureus*) bacteria and the representative fungus species (*Chaetomium globosum*, *Candida albicans*). The results of the antibacterial and antifungal activity test according to the EN-ISO 20645:2006 [71] and the EN 14119:2005 [72] standards are presented in Table 8 and Table 9, and the images are shown in Figure 6, Figure 7 and Figure 8.

**Figure 9:**
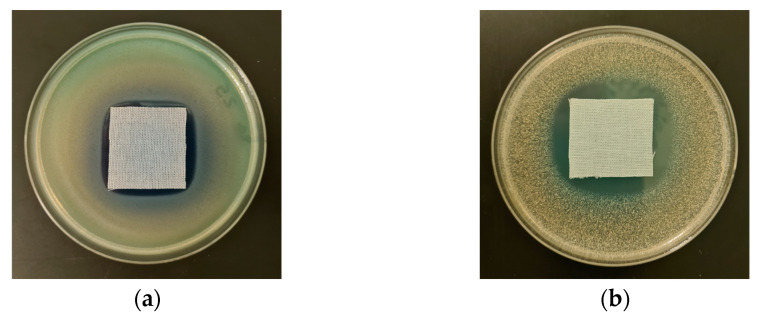
Tests of antimicrobial activity of COT-Cu^(2+)^(0.30) against *P. aeruginosa* (**a**) and *S. aureus* (**b**)—inhibition zones of bacterial growth in Petri dishes according to the EN-ISO 20645:2006 standard.

Control samples–the unmodified cotton material (COT) and the material modified with alginic acid sodium salt (COT-ALG^(−)^Na^(+)^) exhibited strong growth of bacterial and fungal colonies covering the entire surface of the samples placed on Petri dishes (Figure 6, Figure 7 and Figure 8). Cotton material functionalized by copper (COT-Cu^(2+)^) and copper/alginate complex (COT-ALG^(−)^Cu^(2+)^) showed an inhibitory effect against *E. coli*, *S. aureus,* and *P. aeruginosa* bacteria and fungus species expressed by zones of inhibition, with no visible growth on/under the samples (Table 8 and Table 9, Figure 6, Figure 7, Figure 8 and Figure 9).

The obtained results show that the simultaneous modification with copper and alginate allows for a superior antibacterial effect against the *E. coli* bacteria. For samples modified with the same solution of CuCl_2_, i.e., 5% and 10%, the inhibition zones were higher in the case of cotton–alginate composites (3 mm and 5 mm, respectively) than for cotton samples (2 mm and 3 mm, respectively). At the same time, no significant difference in the antimicrobial activity was observed for other tested microorganisms.

The results obtained in accordance with the EN-ISO 20645:2006 and EN 14119:2005 standards [71,72] confirmed the antimicrobial protection of cotton–alginate–copper composite materials (COT-ALG^(−)^Cu^(2+)^) against various type of microorganisms. This is in agreement with our previous work on the antimicrobial properties of alginate–copper complexes [40]. These results are comparable also with Grace et al.’s data [73].

#### 2.3.2. Blood Plasma Clotting: aPTT and PT

**Figure 10:**
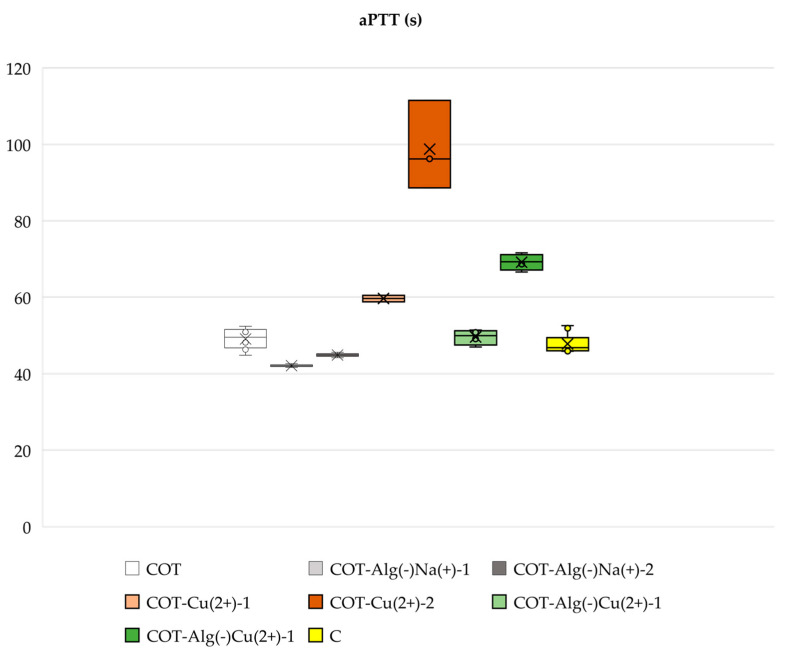
Effect of studied alginate composites on aPTT: COT; COT-ALG^(−)^Na^(+)^(1%)1; COT-ALG^(−)^Na^(+)^(9.7%); COT-Cu^(2+)^(0.16); COT-Cu^(2+)^(0.30); COT-ALG^(−)^Cu^(2+)^(0.15); COT-ALG^(−)^Cu^(2+)^(0.28); and C—control sample: plasma not exposed to composites. Results are presented as mean (×), median (horizontal line), range (bars), and interquartile range (box).

**Figure 11:**
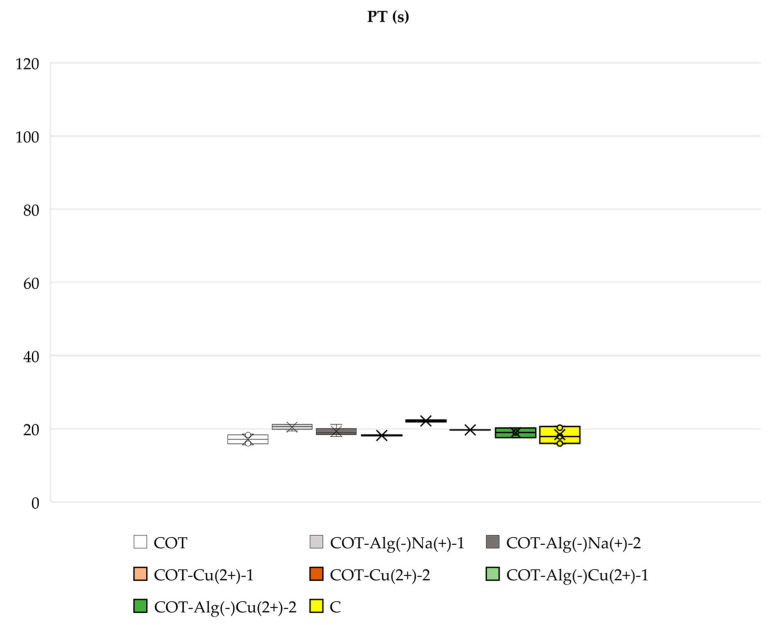
Effect of studied alginate composites on PT: COT; COT-ALG^(−)^Na^(+)^(1%); COT-ALG^(−)^Na^(+)^(9.7%); COT-Cu^(2+)^(0.16); COT-Cu^(2+)^(0.30); COT-ALG^(−)^Cu^(2+)^(0.15); COT-ALG^(−)^Cu^(2+)^(0.28); and C—control sample: plasma not exposed to composites. Results are presented as mean (×), median (horizontal line), range (bars), and interquartile range (box).

The studied composites (COT-ALG^(−)^Na^(+)^ and COT-ALG^(−)^Cu^(2+)^) did not have a significant noxious effect on blood plasma coagulation (PT and aPTT), except COT-ALG^(−)^Cu^(2+)^(0.28) (Cu concentration: 17.51 g/kg), where only aPTT was extended. The materials without alginate (COT-Cu^(2+)^) had a much more prolonged aPTT with increasing copper concentration; however, no effect on PT was observed (Figure 10 and Figure 11). Copper in the samples led to some inhibition of contact clotting activation in human blood plasma, as indicated by the prolongation of aPTT, but this effect disappeared in the presence of the alginate at the lower copper concentration, and even at the higher copper concentration, the inhibition was still lower than in the absence of the alginate. There was no effect on PT, regardless of the presence or absence of the alginate, so only contact factors (XI, XII, HK) were probably adsorbed on Cu^2+^ ions, which contributed to a decrease in their concentration in plasma and thus the effect of aPTT prolongation, but alginate abolished this effect. There was no increased activation of the coagulation cascade in the extrinsic pathway; therefore, the remaining elements of both pathways and coagulation cascades were not affected. This conclusion is also justified in light of the few studies on the influence of transition metals on contact proteins, including examining copper, nickel, cobalt, and zinc cations in the second oxidation state enclosed in liposomes, which showed the influence on aPTT and the absorption of factors XI, XII, and HK in human plasma [74,75]. Such properties of copper may therefore have an adverse effect on blood coagulation when used for the production of dressing materials, despite its beneficial antimicrobial properties. Regardless of this phenomenon, other material modifications with alginate and copper together (in relatively low concentrations of Cu (COT-ALG^(−)^Cu^(2+)^(0.15)) had no negative influence on coagulation, so alginate might be used as a protective component of the composite, thus eliminating the harmful effect on the internal coagulation pathway. Such novel composite materials could potentially be used as neutral for blood clotting and antibacterial dressing materials, taking into account their favorable antibacterial copper-derived properties examined in this study and the alginate layer balancing the blood clotting.

## 3. Conclusions

Antimicrobials neutral for blood clotting wound dressing composites could help to solve the contemporaneous challenges of antimicrobial regenerative medicine, especially those related to antibiotic resistance during wound dressing and care. In this study, we have developed and characterized composite materials consisting of: alginic acid sodium salt, cotton, and copper (II) with different selected concentrations of cations of the tested metal. The cotton–alginate–copper composites were prepared through a two-step, dip-coating procedure. The chemical and structural characterizations of the alginate composites were obtained through Fourier Transform Infrared Spectroscopy (FTIR), Flame Atomic Absorption Spectrometry (FAAS), Specific Surface Area (BET), determination of the air filtration parameters, and tensile strength measurements. The obtained materials exhibited antimicrobial in vitro activity against representative Gram-positive/negative bacteria and representative fungus species. The evaluation of the biochemical properties of the new composites was carried out in the area of blood plasma coagulation processes, including: activated partial thromboplastin time (aPTT), prothrombin time (PT), and thrombin time (TT). The biological results showed that complexes of Cu–alginate with antimicrobial properties at appropriately selected Cu^2+^ concentrations had no significant effect on blood plasma coagulation processes, but they still exhibited antimicrobial activity, in contrast to the material based only on cotton and copper, which showed unfavorable properties prolonging clotting in the intrinsic pathway demonstrated by the extension of aPTT. From this point of view, the obtained cotton–alginate–copper composites might be promising materials for biomedical applications, e.g., wound dressing. The great advantage of the cotton–alginate–copper materials in terms of implementation is the low cost and high availability of its components, and the uncomplicated production process. For future clinical applications, it is critical to test the antimicrobial cotton–alginate–copper composites not only against clinically isolated microbes, but also against multidrug-resistant strains, and to evaluate the in vitro and in vivo biocompatibility, as well as to assess blood coagulation and wound care by applying in vivo mammalian model systems [66]. Negligible moderate neutrality for blood clotting and antibacterial toxicity of the new composites might find great potential in the dressing and healing of wounds with simultaneous prevention and treatment of infections.

## 4. Materials and Methods

### 4.1. Materials

- Alginic acid sodium salt (CAS Number 9005-38-3; molecular weight: 120,000–190,000 g/moL; mannuronic acid to guluronic acid–M/G ratio: 1.56) from Millipore Sigma (St. Louis, MO, USA) was used for the surface modification of polymer nonwovens;
- Medical fabric with a plain weave, qualitative composition: cotton (100% *w*/*w*), weight: 200 g/m^2^ (Andropol S.A., Andrychów, Poland);
- Copper(II) chloride, CuCl_2_, 97% (CAS Number: 7447-39-4) from Millipore Sigma (St. Louis, MO, USA) was used for surface modification of the nonwoven composite;
- Bacterial strains: *E. coli* (ATCC 25922), *S. aureus* (ATCC 6538), and *P. aeruginosa* (ATCC 27853) were purchased from Microbiologics (St. Cloud, MN, USA);
- Fungal strains: *C. albicans* (ATCC 10231) and *Ch. globosum* (ATCC 6205) were purchased from Microbiologics (St. Cloud, MN, USA).
- Standard human blood plasma lyophilizates and clotting times reagents (Dia-PTT, Dia-PT, 0.025 M CaCl_2_ solution) were obtained from a vendor (Diagon Kft, Budapest, Hungary) and prepared according to the manufacturer’s instructions for measurements (K-3002 OPTIC coagulometers, KSELMED^®^, Grudziądz, Poland).

### 4.2. Methods

#### 4.2.1. Chemical and Structural Characterization

##### Attenuated Total Reflection–Fourier-Transform Infrared Spectroscopy (ATR-FTIR)

The chemical structure of the samples was assessed using ATR-FTIR spectroscopy in the range of 400–4000 cm^−1^ using a spectrometer (Jasco’s 4200 (Tokyo, Japan)) with the ATR attachment Pike Gladi ATR (Cottonwood, AZ, USA).

##### Flame Atomic Absorption Spectroscopy (FAAS)

Determination of the copper content in alginate composites was assessed through the FAAS method in a similar way to that described earlier [35,37]. The sample was mineralized using a single-module Magnum II microwave mineralizer from Ertec (Wroclaw, Poland). The determination of the copper (II) ions was performed through atomic absorption spectrometry with flame excitation using a Thermo Scientific Thermo Solar M6 spectrometer (LabWrench, Midland, ON, Canada). The measurements were made in triplicate, and the results were presented as a mean value.

##### Microscopy Analysis

The morphology of the investigated samples was assessed using a VHX-7000N digital microscope (Keyence, Japan). The applied magnification was equal to ×500 and ×2500. The microscope was equipped with the EA-300 laser-based elemental analyzer, which uses laser-induced breakdown spectroscopy (LIBS) to assess the elemental composition of the sample.

##### Specific Surface Area

The specific surface area was determined through the Brunauer, Emmet, and Teller method (BET). Measurements were carried out using the Autosorb-1 apparatus (Quantachrome Instruments, Boynton Beach, FL, USA), with nitrogen as a sorption agent and an adsorption isotherm at 77 K. In each experiment, approximately 1–2 g of a given sample was weighed and used. Prior to the analysis, the samples were dried in 105 °C for 24 h and degassed at room temperature. Measurements were made in duplicate, and the results were presented as a mean value.

##### Air Filtration Parameters

Air permeability of the cotton–alginate–copper composites was determined according to the EN ISO 9237:1998 standard [69]. The FX 3300 TEXTEST AG (Klimatest, Poland) permeability tester was used. Air at a pressure of 100 Pascal was passed through a fabric area of 20 cm^2^ diameter for testing.

##### Tensile Testing

Tensile testing of the cotton–alginate–copper composites was carried out in accordance with the EN ISO 10319:2015-08 standard [70]. An Tinius Olsen H50KS (USA) tester was used. The stretching speed was 20 mm/min.

#### 4.2.2. Antimicrobial Activity

The antibacterial activity of the COT-Alg^(−)^Cu^(2+)^ material was assessed according to standard PN-EN ISO 20645:2006 against a representative colony of Gram-negative and Gram-positive bacteria (*E. coli, S. aureus,* and *P. aeruginosa*) [71]. The antifungal activity of the composites was tested according to PN–EN 14119:2005 against *Ch. globosum* (ATCC 6205) and *C. albicans* (ATCC 10231) [72]. All tests were carried out in duplicate.

#### 4.2.3. Activated Partial Thromboplastin Time (aPTT) and Prothrombin Time (PT)

Pieces of the studied fabrics (1 mg square slices) were supplemented with 200 μL plasma samples and vortexed and incubated for 15 min at 37 °C. Control samples that were not exposed to composites were also incubated for 15 min at 37 °C. The aPTT was completed for each sample. First, 50 μL of the plasma sample and 50 μL of the suspension of Dia-PTT were added to a cuvette located in the thermostat of the coagulometer (37 °C) and left for 3 min. The measurements were initiated by inserting 50 μL of 0.025 M CaCl_2_. The PT was then run: plasma (50 μL) was kept warm (37 °C) in the thermostat of the coagulometer for 2 min, and Dia-PT (50 μL) was added to start the measurement.

## Figures and Tables

**Table 1:** Composition of the alginate dip-coating solution (%).

Sample Assignments/Name	Mixture Components of Film-Forming Material (%)			
	Sodium Alginate Solution		Copper(II) Chloride Solutions	
	0.5%	5%	5%	10%
COT	−	−	−	−
COT-ALG^(−)^Na^(+)^-1 ^(a)^	+	−	−	−
COT-ALG^(−)^Na^(+)^-2 ^(a)^	−	+	−	−
COT-ALG^(−)^Cu^(2+)^-1	+	−	+	−
COT-ALG^(−)^Cu^(2+)^-2	+	−	−	+
COT-Cu^(2+)^-1	−	−	+	−
COT-Cu^(2+)^-2	−	−	−	+

^(a)^ Gravimetric analysis of COT-ALG^(−)^Na^(+)^ samples led to estimation that COT-ALG^(−)^Na^(+)^-1 contained 1% alginate and COT-ALG^(−)^Na^(+)^-2 contained 9.7% alginate. These composites were coded in the text as COT-ALG^(−)^Na^(+)^(1%) and COT-ALG^(−)^Na^(+)^ (9.7%), respectively.

**Table 2:** Characteristic FTIR bands ν (cm^−1^) determined for COT nonwoven, alginate salts ALG^(−)^Na^(+)^, and COT-ALG^(−)^Cu^(2+)^(0.15) composite.

Lit: Cellulose		Lit: ALG^(−)^Na^(+)^		Sample: COT		Sample: COT-ALG^(−)^Cu^(2+)^	
(ν/cm^−1^)	Assign./(Ref.) [57]	Lit. Bands ν (cm^−1^) [58,59,60,61,62]	Assign./(Ref.) [58]	ν (cm^−1^)	Int./^(a)^	ν (cm^−1^)	Int./^(a)^
3300	ν_s_ O-H	3350 ± 350	ν_s_ O-H	3400	0.04/^(b)^	3292	0.11
2893	ν_s_ C-H	2926 ± 1	ν_s_ C-H	2887	0.05	2900	0.09
		1650	ν_as_ COO^−^				
		1614	ν_as_ COO^−^			1590	0.13
1429	γ O-H	1417	δ C–H, ν_s_ COO^−^	1428	0.02	1417	0.12
1370	γ CH_2_			1308	0.03	1305	0.12
1120	ν_as_ C-O-C	1301 (ms)	δ C–H	1150	0.03	1150	0.1
		1096 (s)	ν C–O, ν C–C, δ C–C–O			1094	0.15
1060	ν_s_ C-C-O	1124 (s)	ν C–O, ν C–C, δ C–C–C, ν_as_ C–O–C/^(c)^				
1000	ν C-C	1034 (vs)	ν_as_ C–O–C/^(c)^, ν C–O/^(c)^, ν C–C	1000	0.15	1007	0.23
893	ν C^1^			892	0.07		
		776 (w)	rb, δ C–C–H, δ C–C–O				
		703(ms)	rb				
			δω O–H				
670	ω O–H	826 (ms)	δ C–O–C/^(c)^, δ C–C–C, δ C–C–O/^(c)^ δ C–C–H, δω O–H	684	0.06	673	0.19

^(a)^ Band assignment: ν—stretching vibration; δ—deformation; sh—shoulder; s—symmetric; as—asymmetric; r—rocking; γ—bending mode; ω—wagging; rb—ring breathing. ^(b)^ Band intensity: the bands with absorbance values ≥0.005 were listed and subsequently approximated to a second decimal place. For data from the literature: s—strong, vs—very strong, ms—medium strong, w—weak. ^(c)^ Glycosidic linkage.

**Table 3:** Copper concentration in alginate composite samples.

Sample	Cu Concentration			Ultimate Forms of Samples Abbreviations
	(g/kg)	% (g/100 g)	Ml (mol/kg)	
COT	0.002	0.0002	0.00003	
COT-ALG^(−)^Na^(+)^-1	0.002	0.0002	0.00003	COT-ALG^(−)^Na^(+)^(1%)
COT-ALG^(−)^Na^(+)^-2	0.002	0.0002	0.00003	COT-ALG^(−)^Na^(+)^(9.7%)
COT-Cu^(2+)^-1	10.06	1.01	0.16	COT-Cu^(2+)^(0.16)
COT-Cu^(2+)^-2	19.19	1.92	0.30	COT-Cu^(2+)^(0.30)
COT-ALG^(−)^Cu^(2+)^-(1)	9.35	0.935	0.147	COT-ALG^(−)^Cu^(2+)^(0.15)
COT-ALG^(−)^Cu^(2+)^-(2)	17.51	1.75	0.276	COT-Alg^(−)^Cu^(2+)^(0.28)

COT-ALG^(−)^Na^(+)^(%) samples–% percentage concentration of alginate in corresponding COT-ALG^(−)^Na^(+)^ composites (1% or 9.7%); COT-ALG^(−)^Cu^(2+)^(Ml) samples–Ml molal concentration of copper in corresponding COT-ALG^(−)^Cu^(2+)^(Ml) composites (0.15 molal or 0.28 molal). The results have been measured in triplicate and are presented as a mean value with ± deviation equal to approximately 2%.

**Table 4:** Elemental compositions of the composites investigated based on the laser-based elemental analyzer (SCLA).

Sample	Analysis	Quantitative Content of Elements of Samples (wt.%)			
		C	H	O	Cu
COT	SCLA	35	9	56	-
	BCEA	44.4	6.22		
	CEC ^(a)^	(44.4)	(6.17)	(49.4)	
ALG^(−)^Na^(+)^ × 2H_2_O	CEC ^(a)^	(30.8)	(4.70)	(54.7)	
ALG^(−)^Na^(+)^ × *n*H_2_O [66]	BCEA	29.	4.5		
COT-ALG^(−)^Cu^(2+)^(0.15) ^(c)^	SCLA	33	9	54	4
	BCEA				0.94 ^(b)^
COT-ALG^(−)^Cu^(2+)^(0.28) ^(c)^	SCLA	28	9	50	13
	BCEA				1.75 ^(b)^

Methods: BCEA—Bulk Composition Elemental Analysis based on Combustion Elemental Analysis; CEC—Calculated Elemental Composition (data in brackets); SCLA—Surface Composition Laser Analysis (the results have been measured in duplicate and are presented as a mean value with ± deviation equal to approximately 2%); ^(a)^ CEC data for cellulose (C_6_H_10_O_5_)*_n_* (*n* × 162)) and/or sodium alginate (C_6_H_7_O_6_Na × 2H_2_O)*_n_* (*n* × 234), respectively; ^(b)^ Data taken from Table 3; ^(c)^ COT-ALG^(−)^Cu^(2+)^(Ml) samples–Ml—molal concentration of copper in corresponding COT-ALG^(−)^Cu^(2+)^ composites (0.15 molal or 0.28 molal).

**Table 5:** The specific surface area and total pore volume for the unmodified cotton sample (COT) and cotton–alginate composites (COT-ALG^(−)^Na^(+)^ and COT-ALG^(−)^Cu^(2+)^).

Sample Name	Specific Surface Area (m^2^/g)	Total Pore Volume (cm^3^/g)
COT	0.7359	7.408 × 10^−3^
COT-ALG^(−)^Na^(+)^(1%)	0.6191	3.095 × 10^−3^
COT-ALG^(−)^Na^(+)^(9.7%)	0.5577	2.840 × 10^−3^
COT-ALG^(−)^Cu^(2+)^(0.15)	0.6042	3.033 × 10^−3^
COT-ALG^(−)^Cu^(2+)^(0.28)	0.5931	2.998 × 10^−3^

The results have been measured in duplicate and are presented as a mean value with ± deviation equal to approximately 2%.

**Table 6:** The air flow resistance of COT, COT-ALG^(−)^Na^(+)^, and COT-ALG^(−)^Cu^(2+)^ sample according to the EN ISO 9237: 1998 standard [69].

Parameter	COT	COT-ALG^(−)^ Na^(+)^(1%)	COT-ALG^(−)^ Na^(+)^(9.7%)	COT-ALG^(−)^Cu^(2+)^(0.15)	COT-ALG^(−)^Cu^(2+)^(0.28)
Average air permeability (mm/s)	810 ± 7	278 ± 35	39.3 ± 3.8	283 ± 41	286 ± 35

The results have been measured in duplicate and are presented as the mean value with ± deviation of approximately 2%.

**Table 7:** Tensile strength test of COT, COT-Alg^(−)^Na^(+)^, and COT-Alg^(−)^Cu^(2+)^ sample according to the EN ISO 10319:2015-08 standard [70].

Parameter		COT	COT-ALG^(−)^ Na^(+)^(1%)	COT-ALG^(−)^ Na^(+)^(9.7%)	COT-ALG^(−)^ Cu^(2+)^(0.15)	COT-Alg^(−)^ Cu^(2+)^(0.28)
Tensile strength (N)	(a)	490 ± 10	510 ± 30	580 ± 10	513 ± 25	515 ± 20
	(b)	380 ± 10	370 ± 70	410 ± 20	375 ± 50	380 ± 60
Relative elongationat maximum load (%)	(a)	7.6 ± 1.6	11.0 ± 1.5	10.0 ± 1.5	11.0 ± 1.6	11.0 ± 1.5
	(b)	28.0 ± 2.0	34.5 ± 3.5	29.5 ± 3.0	35.1 ± 4.0	33.4 ± 4.3

The results have been measured in duplicate and are presented as the mean value with ± deviation of approximately 2%; (a): parallel direction; (b): transverse direction.

**Table 8:** Results of antimicrobial activity tests of alginate composites on the basis of the EN-ISO 20645:2006 standard [71].

Sample Name	Average Inhibition Zone (mm)		
	*E. coli*	*S. aureus*	*P. aeruginosa*
COT	0	0	0
COT-ALG^(−)^Na^(+)^(1%)	0	0	0
COT-ALG^(−)^Na^(+)^(9.7%)	0	0	0
COT-Cu^(2+)^(0.16)	2	2	2
COT-Cu^(2+)^(0.30)	3	4	4
COT-ALG^(−)^Cu^(2+)^(0.15)	3	2	2
COT-ALG^(−)^Cu^(2+)^(0.28)	5	4	3

Concentration of inoculum (CFU/mL): *E. Coli*—2.1 × 10^8^, *S. Aureus—*1.9 × 10^8^, *Ch. Globosum*—2.0 × 10^6^, *P. aeruginosa*—2.2 × 10^8^.

**Table 9:** Results of antimicrobial activity tests of alginate composites on the basis of the EN 14119: 2005 standard [72].

Sample Name	Average Inhibition Zone (mm)	
	*Ch. globosum*	*C. albicans*
COT	0	0
COT-ALG^(−)^Na^(+)^(1%)	0	0
COT-ALG^(−)^Na^(+)^(9.7%)	0	0
COT-Cu^(2+)^(0.16)	no growth	no growth
COT-Cu^(2+)^(0.30)	no growth	no growth
COT-ALG^(−)^Cu^(2+)^(0.15)	no growth	no growth
COT-ALG^(−)^Cu^(2+)^(0.28)	no growth	no growth

Concentration of inoculum (CFU/mL): *Ch. Globosum*—2.0 × 10^6^, *C. albicans*—0.8 × 10^8^.

## Data Availability

The original data are available on request.
